# Improved Sample Preparation for Untargeted Metabolomics Profiling of Escherichia coli

**DOI:** 10.1128/Spectrum.00625-21

**Published:** 2021-10-06

**Authors:** Dongyang Ye, Xiaowei Li, Chengfei Wang, Saiwa Liu, Liang Zhao, Jingjing Du, Jian Xu, Jing Li, Lu Tian, Xi Xia

**Affiliations:** a College of Veterinary Medicine, China Agricultural University, Beijing, China; Georgia Institute of Technology

**Keywords:** metabolomics, quenching, ethylene glycol, *Escherichia coli*, antibiotic

## Abstract

Metabolomics is a powerful tool that can systematically describe global changes in the metabolome of microbes, thus improving our understanding of the mechanisms of action of antibiotics and facilitating the development of next-generation antibacterial therapies. However, current sample preparation methods are not efficient or reliable for studying the effects of antibiotics on microbes. In the present study, we reported a novel sample preparation approach using cold methanol/ethylene glycol for quenching Escherichia coli, thus overcoming the loss of intracellular metabolites caused by cell membrane damage. After evaluating the extraction efficiency of several extraction methods, we employed the optimized workflow to profile the metabolome of E. coli exposed to cephalexin. In doing so, we proved the utility of the proposed approach and provided insights into the comprehensive metabolic alterations associated with antibiotic treatment.

**IMPORTANCE** The emergence and global spread of multidrug-resistant bacteria and genes are a global problem. It is critical to understand the interactions between antibiotics and bacteria and find alternative treatments for infections when we are moving closer to a postantibiotic era. It has been demonstrated that the bacterial metabolic environment plays an important role in the modulation of antibiotic susceptibility and efficacy. In the present study, we proposed a novel metabolomic approach for intracellular metabolite profiling of E. coli, which can be used to investigate the metabolite alterations of bacteria caused by antibiotic treatment. Further understanding of antibiotic-induced perturbations of bacterial metabolism would facilitate the discovery of new therapeutic targets and pathways.

## INTRODUCTION

The emergence and global spread of multidrug-resistant bacteria and genes that reduce antibiotic efficacy pose serious threats to human health (1). The development of novel antibiotics is both costly and complex, so it is particularly important to develop alternative therapies and further study the mechanisms of action of existing drugs (2). Metabolic states of bacteria may play a vital role in the modulation of antibiotic susceptibility and efficacy (3). Moreover, exogenous metabolites can restore the susceptibility of drug-resistant bacteria to kanamycin (4). Metabolomics is a powerful tool for studying microbial metabolism, enabling systematic identification and quantitation of global changes in the metabolome. Highly reliable, reproducible, and efficient sample preparation methods are essential to obtain both accurate and complete microbial metabolite data (5), which will enhance our understanding of the metabolic perturbations induced by antibiotics (6).

The turnover of both primary and secondary metabolism can be extremely rapid, in the range of subseconds to tens of seconds (7). For instance, cytosolic glucose is converted at approximately 1 mM/s in Saccharomyces cerevisiae (8), while the turnover rates of ATP and ADP are between 1.5 and 2.0 mM/s (9). During the process of sample preparation, cell quenching is crucial for the rapid inhibition of intracellular enzyme activity and the extraction of a real “snapshot” of intracellular metabolites. A frequently used quenching solvent is cold 60% methanol aqueous solution, which has been widely applied in different microorganisms, including Escherichia coli (10, 11), Lactococcus lactis (12), Lactobacillus plantarum (13), Lactobacillus bulgaricus (14), Penicillium chrysogenum (15), Aspergillus niger (16, 17), Pichia pastoris (18), and S. cerevisiae (19). However, when cold methanol/water or pure methanol quenching was used, intracellular metabolites leaked from bacterial cells, making accurate determination of their intracellular levels challenging to achieve (12). As an alternative, a fast filtration system combined with quenching/extraction has been proposed as an effective strategy to reduce the loss of intracellular metabolites (20). However, performing manual fast filtration and clogging of filters are inevitable drawbacks of this approach (21, 22). Alternative quenching strategies that reduce the leakage of intracellular metabolites and stabilize bacterial cells have been tested with limited success, including using different methanol concentrations (14), addition of buffering solutions (13, 23, 24), and changing the ratios of bacterial culture to quenching solvents.

The extraction of intracellular metabolites after fast filtration or quenching is also essential for the successful preparation of microbial metabolome samples. Ideally, intracellular metabolites should be nonselectively and completely extracted without degradation and conversion during the extraction process (25). Different extraction solvents have been reported, such as an acetonitrile-methanol-water mixture (26–28), pure methanol (29, 30), and methanol/water, but each has its respective limitations. For instance, a mixture of cold methanol-chloroform-water was not suggested for extracting metabolites that can be oxidized (31, 32), and the ratio of cold methanol/water could impact the effectiveness of extraction.

In the present study, we established a robust untargeted metabolomics method for investigations into perturbations of bacterial metabolism by antibiotics based on cold methanol/ethylene glycol quenching, boiling ethanol/water extraction, and ultra-high-performance liquid chromatography combined with time of flight mass spectrometry (UHPLC-TOF/MS) detection. The proposed method prevented substantial loss of intracellular metabolites during quenching of E. coli, resulting in high quenching efficiency, improved cell membrane integrity, and excellent extraction reproducibility. We then applied our novel workflow to study the effects of cephalexin on the metabolome of E. coli. We expect that our workflow will contribute to further investigations into the effects of antibiotics on microbial metabolism, enabling better understanding of the mechanisms by which bacteria evade antibiotics.

## RESULTS AND DISCUSSION

### Ethylene glycol-based quenching obtained higher metabolite abundances.

**FIG 1:**
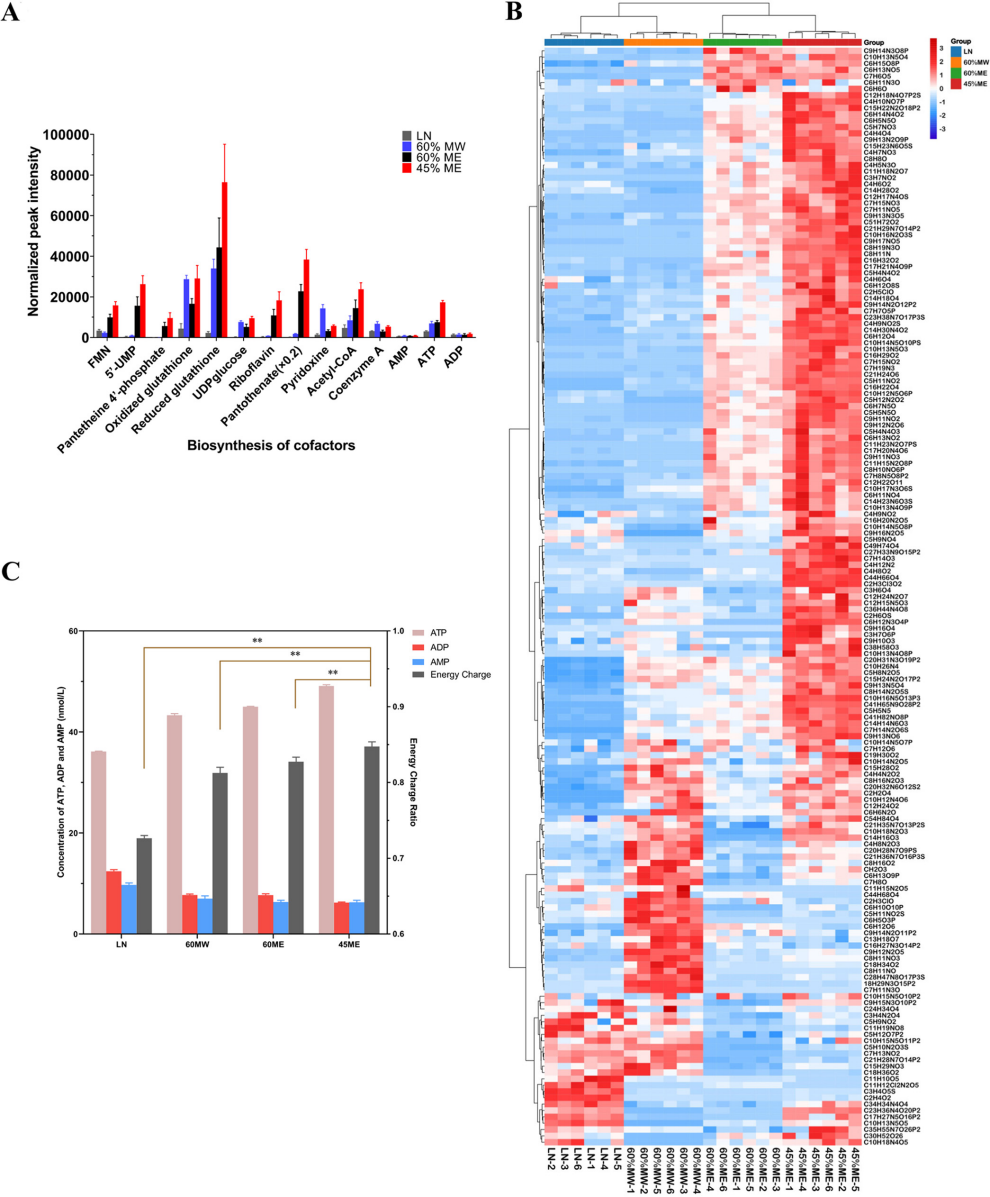
Quenching solvent optimization. (A) Abundance of representative identified metabolites using different quenching methods. (B) Hierarchical clustering analysis of representative detectable features of E. coli obtained using LN, 60% MW, 60% ME, and 45% ME quenching approaches. (C) Comparison of intracellular ATP, ADP, AMP, and energy charge of E. coli obtained following LN, 60% MW, 60% ME, and 45% ME quenching.

Bacteria possess the most vigorous enzyme systems and metabolic activity, the highest proportion of living cells, and the most stable physical and chemical properties during the mid-exponential phase (33). We first obtained growth curves of E. coli and determined that the mid-exponential phase was at an approximate optical density at 600 nm (OD_600_) ≈ 0.35 (see Fig. S1 in the supplemental material). Accordingly, all bacterial cultures were subsequently harvested at OD_600_ ≈ 0.35. Methanol/water (MW) (60:40, vol/vol) (60% MW) and liquid nitrogen (LN) are commonly used quenching solvents for the preparation of microbial metabolomics samples (31, 34). In the present study, four quenching approaches, including LN, 60% MW, 60% methanol/ethylene glycol (ME), and 45% ME, were compared to evaluate their quenching efficiency. Considering many detected peaks are adducts, fragments, isotopic ions, or background ions (35), we evaluated the performance of different quenching approaches via the comparison of the abundance of representative metabolites. Most identified metabolites exhibited the highest intensity after quenching using 45% ME (Fig. 1A; see also Fig. S2 in the supplemental material). Representative detectable features using all four quenching strategies, with good abundance and repeatability, were further analyzed. The high-abundance features were distributed differently between the 60% MW and 60% ME/45% ME quenching groups, indicating that different quenching solvents may cause different types of metabolites to leak. According to hierarchical clustering analysis, the feature profiles of the 45% ME and 60% ME quenching groups were similar, while those of the 60% MW and LN quenching groups were similar (Fig. 1B). The 45% ME quenching group had the largest proportion of high-abundance putative metabolites (approximately 77%). The overall abundance of features in the 60% ME group was lower than that of the 45% ME group, indicating that a higher proportion of ethylene glycol may prevent the release of intracellular metabolites. The lowest abundance of features was observed in the LN quenching group, which may be a result of the leakage of intracellular metabolites. Furthermore, the six replicates of the 45% ME and 60% ME quenching groups had higher intragroup feature profile similarity than the other two groups. In addition to the solvents used, the ratio of microbial culture (37°C) to quenching solvent can affect quenching by affecting the temperature (36). After quenching at a 1:3 ratio (sample, quenching solvent), the temperatures of samples were close to −13°C, whereas quenching at a 1:2 ratio resulted in temperatures of samples close to 0°C. Only a 1:4 ratio of sample to quenching solvent could achieve a quenching temperature below −25°C and thus guaranteed the inactivation of metabolism-related enzymes to inhibit microbial metabolic activities (see Fig. S3 in the supplemental material).

Bacterial cells are more likely to leak intracellular metabolites than yeast and filamentous fungal cells during quenching (37). Finding quenching agents that cause less damage to the cell membrane, and thus less leakage of intracellular metabolites, is vital for successful pretreatment methods in microbial metabolomics. Glycerol could protect freeze-dried lactic acid bacteria through inhibiting excess dehydration, reducing salt toxicity, and preventing ice crystal formation (38). A quenching method for microbial cell cultures based on cold glycerol-saline solution has been reported and could significantly prevent intracellular metabolite leakage (36). However, a small amount of glycerol attaches to the centrifuged cell pellets and is difficult to remove because of the high viscosity, boiling point, and polarity of glycerol, resulting in increased column pressure and negatively affecting the measurement of metabolic profiles (14, 39, 40). Ethylene glycol is another cryoprotectant with properties similar to glycerol’s, but with a lower viscosity and boiling point, and was adopted in the present study as a novel quenching agent. Ethylene glycol can stabilize frozen proteins, preventing denaturation during quenching. As a cryoprotectant, ethylene glycol has been used in many biomedical fields, such as limbal stem cell preservation (41) and vitrification of embryos and oocytes in livestock (42), which also showed low cytotoxicity even at high concentrations (43, 44). In a preliminary study, we found 45% ME became a viscous liquid at −60°C, but this property was absent at higher temperatures. Ethylene glycol has a lower boiling point (197.3°C) than glycerol (290°C), and vacuum drying during sample preparation could easily remove the residual ethylene glycol. Therefore, we quenched bacteria at −60°C to ensure the viscosity of ethylene glycol, protect the cell membranes, and prevent leaking of internal metabolites. The use of ethylene glycol as a component of the quenching agent, in particular, 45% ME, could successfully quench bacterial cells in culture.

### Ethylene glycol-based quenching maintained at a high energy charge level.

Energy charge (EC) describes the relationship among ADP, ATP, and AMP in cells, reflecting the energy state of biological systems, and can be used to measure the inactivation of cell metabolism (13). Most bacterial cells maintain an EC of between 0.8 and 0.9 during the exponential phase (45, 46), including Bacillus subtilis, S. cerevisiae, or L. lactis (47–50). Furthermore, changing EC values are thought to be an important factor to trigger metabolic reactions (51), reflecting starvation (45) and diverse pexophagy modes (51). Different quenching agents affect the EC of bacterial cells (20, 52). A homeostasis EC (0.8∼0.9) after quenching reflects the initial metabolic state of cells, which is a precondition for a metabolic sample. Hence, the EC is a vital physiological indictor of cellular metabolic activity and can be used to evaluate quenching efficiency during sample preparation.

EC is maintained at a high level if the metabolism of microorganisms is inactivated rapidly using effective quenching solvents. Quenching with 45% ME, 60% ME, and 60% MW were highly effective at inactivating cell metabolism, as the EC values remained within a physiological range, indicating that these methods can effectively inhibit intracellular enzymes and immediately cease cell metabolism (Fig. 1C). Quenching with 60% MW in E. coli was similar to that in *L. plantarum*, resulting in an EC value in the range of 0.771 ± 0.003 ∼ 0.849 ± 0.003 (53). The highest EC was obtained with 45% ME quenching (0.847 ± 0.006) and was significantly different from that of the other groups (60% ME, 60% MW, and LN) (*P *<* 0.05*). In contrast to the other three quenching solvents, quenching with LN resulted in a low EC (0.726 ± 0.003), suggesting that LN is less effective at the deactivation of cellular metabolism. Interestingly, LN possessed the lowest temperature (−196°C) but resulted in the worst quenching efficiency. This may be due to serious damage to the bacterial cell membrane caused by the ultralow temperature in the absence of cryoprotectant, causing the leakage of ATP, ADP, and AMP.

### Ethylene glycol process protective effect on cell membranes.

**FIG 2:**
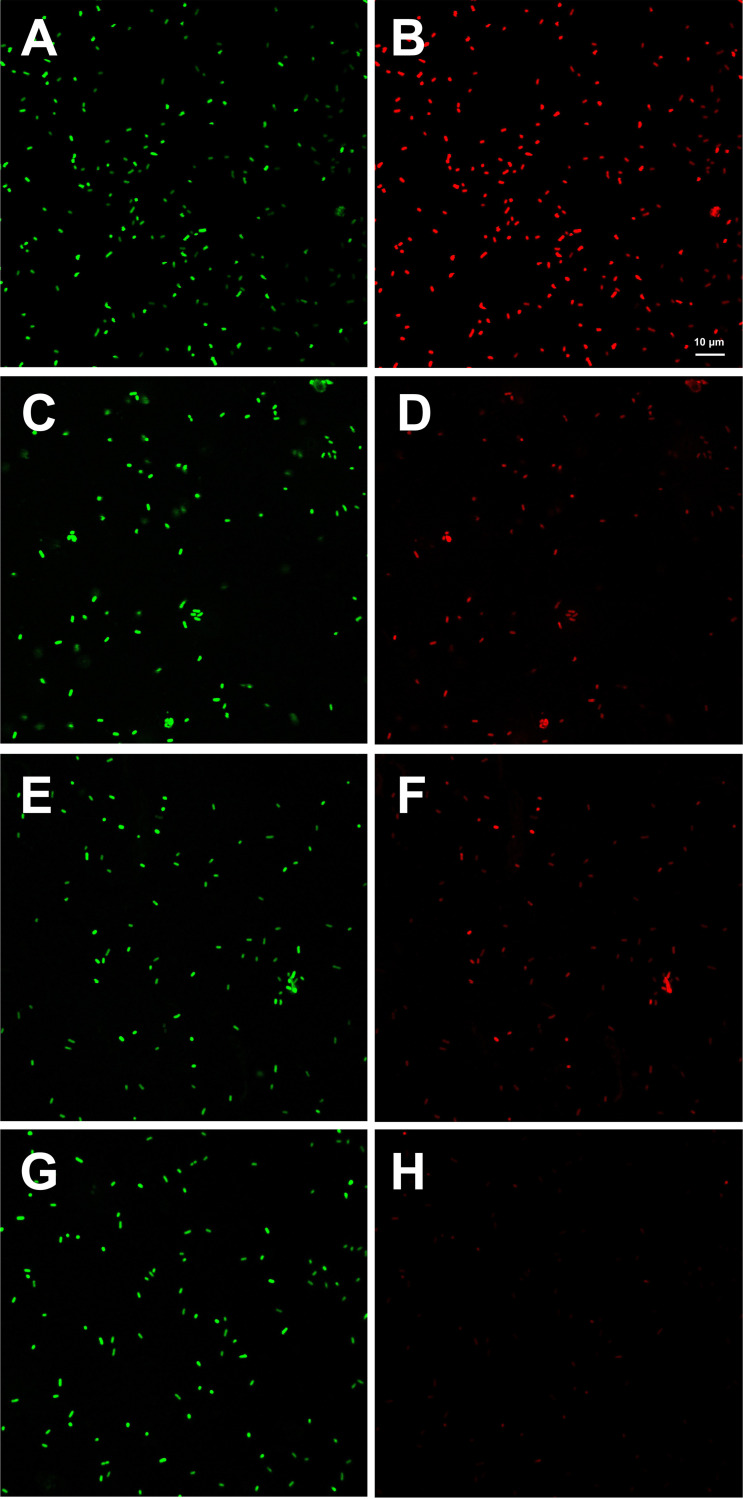
Membrane integrity of E. coli (LN quenching with SYTO 9 staining [A], LN quenching with PI staining [B], 60% MW quenching with SYTO 9 staining [C], 60% MW quenching with PI staining [D], 60% ME quenching with SYTO 9 staining [E], 60% ME quenching with PI staining [F], 45% ME quenching with SYTO 9 staining [G], 45% ME quenching with PI staining [H]).

To investigate cell leakage caused by the different quenching solvents, confocal laser scanning microscopy was performed with two dyes, SYTO 9 and propidium iodide (PI), to evaluate the cell membrane integrity of E. coli after quenching. LN-based quenching resulted in similar intensity of SYTO 9 (Fig. 2A) and PI-stained cells (Fig. 2B). For the 60% MW and 60% ME groups, PI staining (Fig. 2D and F, respectively) showed much lower intensity than that of SYTO 9 staining (Fig. 2C and E, respectively). Compared with SYTO 9 staining (Fig. 2G), few PI-stained cells were observed (Fig. 2H) with 45% ME quenching. SYTO 9 can stain all cells, whereas PI can only permeate and stain cells lacking cell membranes, indicating that LN quenching seriously damaged the cell membrane of E. coli. Although 60% MW and 60% ME also caused damage to the cell membrane of E. coli, they caused less damage than LN quenching. Owing to the low PI staining rate observed, 45% ME quenching caused little damage to the cell membrane of E. coli, indicating that 45% ME can reduce the leakage of intracellular metabolites. The confocal laser scanning microscopy observations agreed with the results of the metabolic profiling after quenching, where the highest abundance of metabolites was observed in the 45% ME group, indicating that an intact cell membrane is critical for obtaining accurate microbial metabolome profiles.

### NaCl washing is necessary during sample preparation.

Without the washing step, extracellular metabolites and culture medium components may still remain on the cell surface after quenching and eventually enter samples, interfering with the analysis of intracellular metabolites. To validate the necessity of the washing step, we spiked [U-^13^C_6_]glucose into the bacterial culture before quenching and compared its concentrations in the samples prepared with and without washing. As shown in Fig. S4 in the supplemental material, the percentage of residual [U-^13^C_6_]glucose after different quenching approaches using NaCl washing was significantly lower than that without washing (*P* < 0.05). Next, we assessed the leakage of representative intracellular metabolites, calculating the percentage of leakage in washing groups or the negative control group versus that in the positive control group (see Fig. S5 in the supplemental material). The highest and lowest percentages of leakage of the tested metabolites were observed in the LN and 45% ME quenching groups, respectively. No leakage of adenine and adenosine was detected following quenching by 45% ME. There were no significant differences in the leakage rates of other metabolites, except riboflavin, between the 45% ME quenching group and negative control group (*P *> 0.05).

### Effect of extraction solvent on metabolic profile.

**FIG 3:**
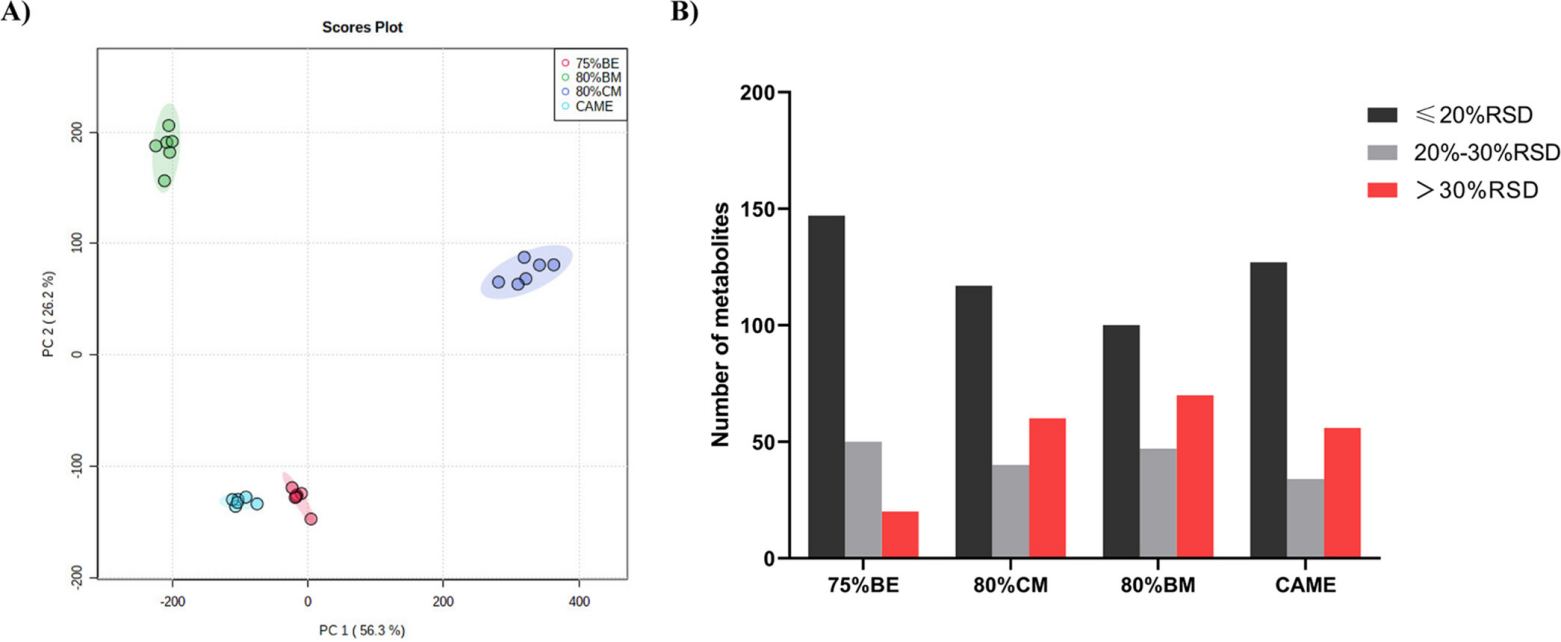
Principal-component analysis (PCA) and relative standard deviation (%RSD) of the intracellular metabolites of E. coli extracted with different solvents. (A) PCA plot. (B) Reproducibility of different extraction solvents expressed as %RSD.

For successful metabolome profiling, intracellular metabolites should be extracted efficiently from cells, and the extraction solvents should not cause any chemical decomposition or degradation of the metabolome. A variety of extractants have been used to obtain microbial intracellular metabolites, and a combination of different ratios of methanol/water with ultrasonic extraction is the main method for E. coli metabolite extraction. In the present study, we focused on total metabolome extraction for untargeted microbial metabolomics and evaluated four different extraction solvents as follows: cold methanol (CM)/water (80:20, vol/vol, 80% CM) at −20°C, boiling methanol (BM)/water (80:20, vol/vol, 80% BM) at 80°C, boiling ethanol (BE)/water (75:25, vol/vol, 75% BE) at 95°C, and cold acetonitrile/methanol/ethanol/water (CAME) (20:20:20:40, vol/vol, at −20°C). The metabolite profiles extracted by 80% CM, 80% BM, 75% BE, and CAME were clearly distinguishable by principal-component analysis (PCA) (Fig. 3A). Next, normalization was performed by subtracting the abundance of each metabolite from the mean of all metabolites and then dividing by the standard deviation. As shown in Fig. S6 in the supplemental material, 75% BE yielded the highest abundances of metabolites overall. The metabolite profiles of 80% CM, 80% BM, and CAME were grouped into one category, whereas that of 75% BE was grouped separately into another class. The reproducibility of metabolite extraction was assessed by calculating the relative standard deviation (%RSD) for the abundances of the metabolites with different extraction solvents. The frequency distributions of %RSD for the four extraction solvents were shown in Fig. 3B. Extraction with 75% BE resulted in the extraction of the most metabolites with ≤20% RSD and 20% to 30% RSD, indicating that this extraction solvent has the best reproducibility among the compared solvents.

Cold methanol-water, boiled ethanol-water (23, 29, 54), cold methanol-chloroform-water mixture (31, 32, 55), acetonitrile-methanol-water mixture (26–28), and other organic solvent-water mixtures have been previously tested for their ability to extract microbial metabolites. Several studies have compared different extraction solvents and optimized the most effective method. However, owing to the diversity of microbial species, different extraction solvents usually have different extraction effects in different species. Moreover, some studies are inconsistent and contradictory under certain conditions (56–59). Therefore, in the present study, we compared different extraction buffers and optimized the method specifically for E. coli. Extraction with the boiled ethanol-water mixture is often used for Gram-positive bacteria. In the present study, 75% BE could obtain a high abundance of metabolic profile with excellent reproducibility, suggesting that this method is also suitable for the extraction of intracellular metabolites from Gram-negative bacteria. This finding is similar to the results reported by Li et al. (60), who compared the extraction effects of pure methanol, ethanol-water, methanol-water, and chloroform-methanol-water on Bacillus licheniformis and found that 75% BE was the most effective solvent.

### Antibiotic treatment induces metabolic alterations.

**FIG 4:**
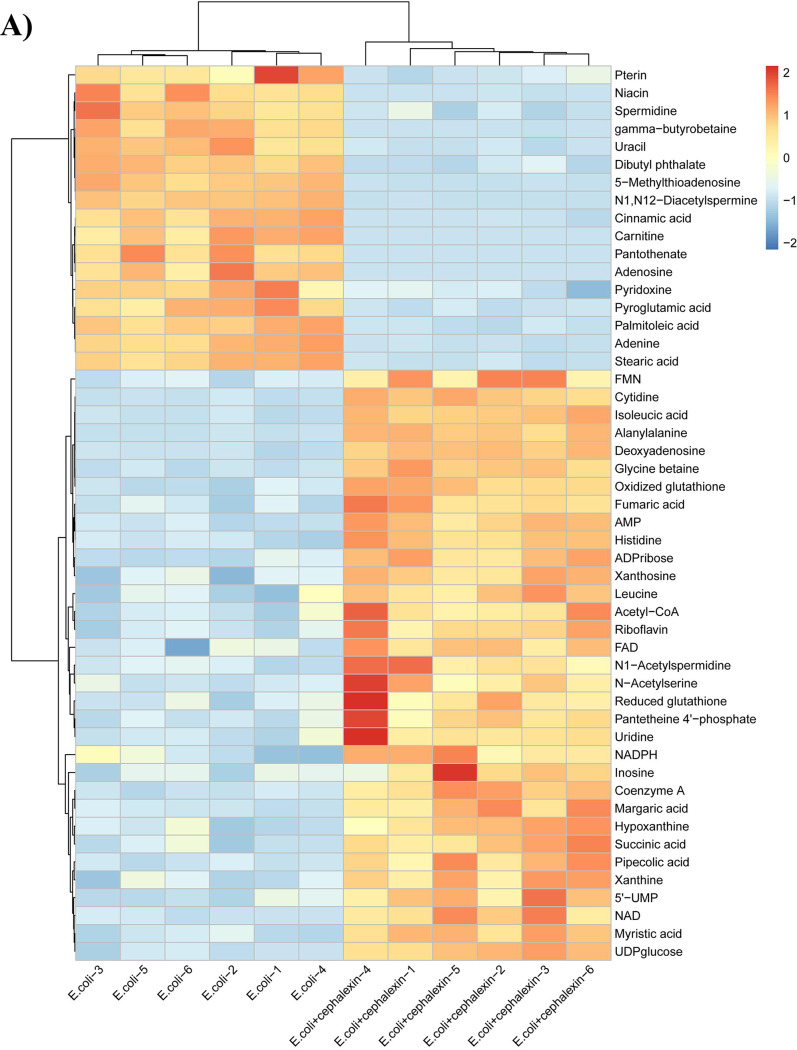

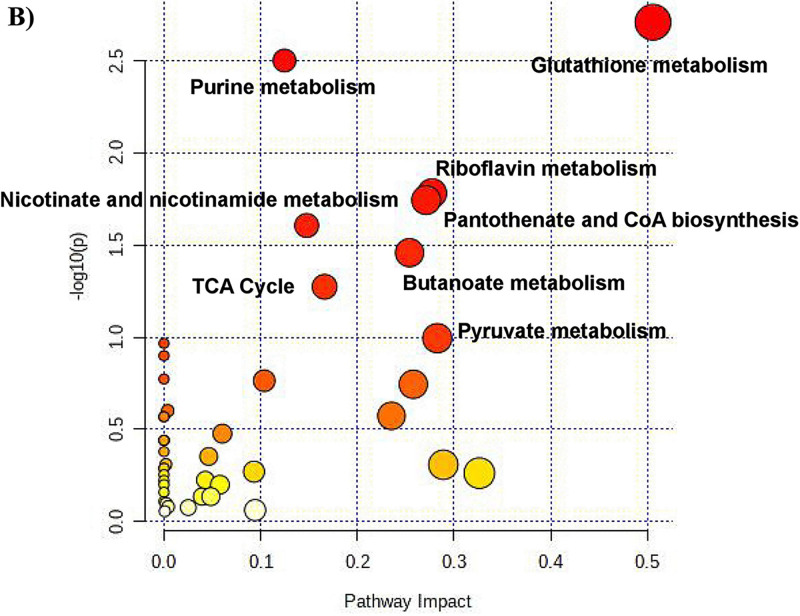
Differential metabolites and pathway analysis of E. coli under antibiotic perturbation. (A) Hierarchical clustering analysis of the differential metabolites. (B) Metabolic pathway analysis of cephalexin-mediated bacterial metabolic perturbations.

Cell death mediated by antibiotics is a complex process that cannot be merely elucidated by the direct interactions between antibiotics and their cellular targets (61–64). A number of studies suggested that cellular metabolic states of bacteria can affect antibiotic efficacy (65, 66). To evaluate the performance of the proposed method for metabolomic profiling, we analyzed the perturbations of metabolism in E. coli after treatment with cephalexin (1.0 μg/ml). A total of 50 differential metabolites, including amino acids, organic acids, adenosines, glutathione, coenzymes, sugars, and nucleotides, were identified (see Fig. S7 in the supplemental material), of which 33 were upregulated and 17 were downregulated (Fig. 4A; see also Table S1 in the supplemental material). The identities of these metabolites were confirmed using standards or tandem mass spectrometry (MS/MS) databases (see Fig. S8 and Table S2 in the supplemental material). Metabolic pathway analysis indicated that the differential metabolites were significantly enriched in the glutathione metabolism, purine metabolism, pantothenate and coenzyme A (CoA) biosynthesis, riboflavin metabolism, and nicotinate and nicotinamide metabolism (Fig. 4B).

**FIG 5:**
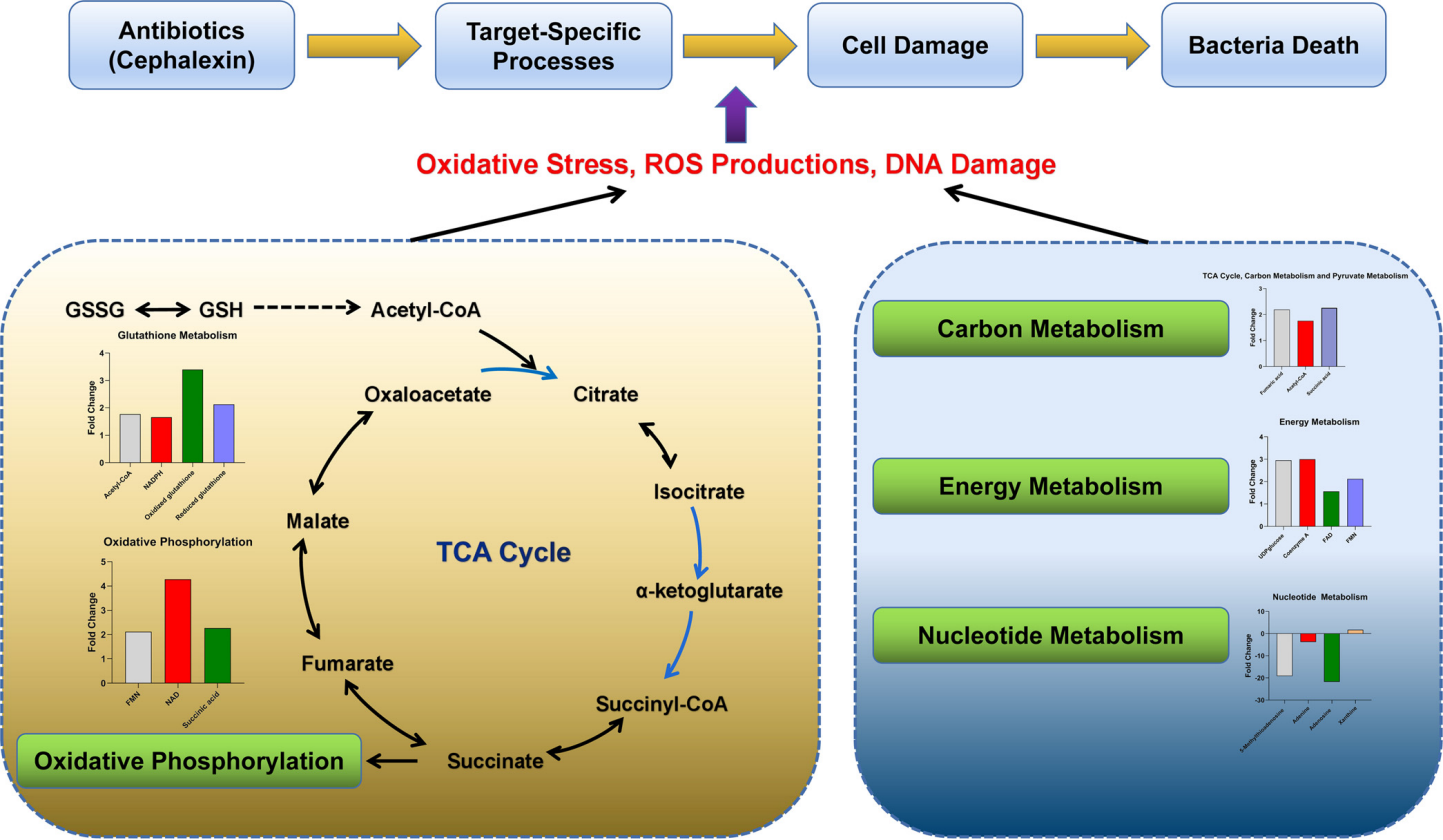
Overall metabolic perturbations associated with cephalexin treatment.

The linkage between the tricarboxylic acid (TCA) cycle and peptidoglycan synthesis has been reported in previous studies (67, 68). The accumulation of intermediate products of TCA and disruption of its homeostasis can affect the synthesis of bacterial peptidoglycan (69). In terms of central carbon metabolism, we found that the most significant changes after cephalexin exposure appeared in the TCA cycle, with acetyl-CoA, fumaric acid, and succinic acid increasing after cephalexin stimulation (see Fig. S9A in the supplemental material). The correlation between the increased levels of fumaric acid and succinic acid and peptidoglycan synthesis should be investigated in further study. We also observed elevated levels of flavin mononucleotide (FMN), NAD, and succinic acid, which indicated that oxidative phosphorylation occurred (Fig. S9B). Together, these results suggest that cephalexin may cause changes in the TCA cycle and oxidative phosphorylation. Pyruvate metabolism was also upregulated (Fig. S9A), which is consistent with a previous report that the pyruvate cycle increases antibiotic efficacy and provides respiratory energy in bacteria (70). These findings are consistent with those of studies on the metabolome of Mycobacterium tuberculosis after antibiotic therapy, demonstrating that the bacterial response was linked to alterations in central energy metabolism and the TCA cycle (71, 72). The energy metabolism was upregulated, with increases in UDPglucose, coenzyme A, flavin adenine dinucleotide, and FMN levels after antibiotic treatment (Fig. S9C). In addition to central carbon metabolism and oxidative phosphorylation, we also observed significant alterations in nucleotide metabolism. Cephalexin treatment resulted in decreased levels of nucleosides, nucleotides, and purine (Fig. S9D), suggesting a decrease in the pool of nucleotide building blocks. This change, together with upregulated xanthine concentrations (marker of purine catabolism), supports the hypothesis that antibiotics may promote the conversion of nucleotide to generate high levels of DNA damage (64). The reduction of the ratio of reduced glutathione (GSH) to oxidized glutathione (GSSG) is generally considered to be an indicator of oxidative stress (73). Accordingly, we detected a greater increase in the concentrations of GSSG than of GSH (Fig. S9E). In addition, riboflavin metabolism was upregulated (Fig. S9F). Changes in the levels of these metabolites indicate that the biosynthesis of GSH increased to counteract the sustained turnover caused by antioxidant activities. Taken together, these results revealed the metabolic changes related to the bactericidal effect of cephalexin (Fig. 5).

## MATERIALS AND METHODS

### Strain and culture conditions.

E. coli ATCC 25922 was inoculated into 1 ml LB broth (1% [wt/vol] tryptone, 0.5% [wt/vol] yeast extract, 1% [wt/vol] NaCl) and cultured overnight at 37°C in a humidity controlled shaking incubator (Shanghai Zhicheng Analytical Instrument Manufacturing Co., Ltd.). The overnight culture of bacteria was inoculated into a baffled flask containing LB broth and agitated at 260 rpm until the OD_600_ indicated that the culture had reached the mid-exponential phase (approximately 0.35).

### Quenching and extraction.

Ten-milliliter aliquots of bacterial cultures were directly transferred to a 100-ml centrifuge tube containing 40 ml of methanol/ethylene glycol (45:55, vol/vol) (45% ME) precooled to −60°C, mixed for 5 s, and then centrifuged at 16,000 × *g* for 10 min at −20°C (Beckman Coulter Inc., USA). The supernatant was discarded, and the pellets were washed twice with 0.85% NaCl and then centrifuged at 16,000 × *g* for 10 min at 4°C. Subsequently, the pellets were transferred to 1.5-ml microfuge tubes and extracted with 500 μl of boiling ethanol/water (75:25, vol/vol) (75% BE) at 95°C for 5 min. The samples were then centrifuged at 30,000 × *g* for 15 min at 4°C, and the supernatants were collected and dried using a SpeedVac (SPD111V; Thermo Fisher Scientific, USA). Finally, the extracts were dissolved in 500 μl of acetonitrile/water (50:50, vol/vol) for UHPLC-TOF/MS analysis. Aliquots (30 μl) from all samples were mixed to prepare the quality control sample. Six biological replicates were prepared and analyzed.

### Quenching efficiency.

Energy charge (EC) was used as an indicator to evaluate the inactivation of cell metabolism. After quenching and extraction, intracellular ADP, ATP, and AMP were quantified using a kit (MSKBio, Wuhan, China), based on enzyme-linked immunosorbent assay by double antibody sandwich method. The OD_450_ was measured, and the concentrations of ATP, ADP, and AMP were calculated using standard curves. The EC was determined as follows: EC = ([ATP] + 0.5[ADP])/([ATP] + [ADP] + [AMP]).

### Confocal laser scanning microscopy.

To evaluate cell membrane integrity after quenching, the cells after quenching were stained with fluorescent dyes SYTO 9 (Invitrogen, Carlsbad, USA) and propidium iodide (PI) (Invitrogen). The quenched cell pellets were washed twice with sterile phosphate-buffered saline (PBS) (Sangon Biotech, Shanghai, China) and resuspended in 2 ml of PBS. Cell suspensions (1 ml) were diluted by adding 20 ml of PBS and incubated at 30°C for 1 h, mixing every 15 min. The incubated cell pellets were washed twice and resuspended in 10 ml of PBS. Equal volumes of SYTO 9 and PI were added to the bacterial suspension at a final concentration of 3 μl/ml, and the mixture was incubated at room temperature in the dark for 15 min. The stained bacterial suspension (5 μl) was pipetted onto a glass slide and covered with a coverslip. The stained, quenched cells were visualized by confocal laser scanning microscopy (Nikon A1R MP, Tokyo, Japan) with a 543-nm He-Ne laser (red channel) and a 488-nm Ar laser (green channel).

### Evaluation of washing with NaCl.

To investigate the necessity of washing with NaCl, [U-^13^C_6_]glucose (Sigma-Aldrich, Saint Louis, USA) was added into the bacterial culture and mixed, and bacteria were quenched immediately and washed twice with 0.85% NaCl. The pellets were resuspended in the 0.85% NaCl solution, the remaining [U-^13^C_6_]glucose was concentrated using C_18_ cartridges, and the concentration was determined using UHPLC-TOF/MS. To evaluate the leakage of intracellular metabolites after quenching caused by NaCl washing, the NaCl washing solutions from different quenching approaches were collected and concentrated using C_18_ cartridges, and the metabolites were analyzed by UHPLC-TOF/MS. The positive control group sample was suspended in 0.85% NaCl and heated in a water bath at 80°C for 20 min. Unquenched bacteria were used as a negative control.

### Antimicrobial perturbations to the E. coli metabolome.

E. coli ATCC 25922 was grown in LB broth at 37°C to an OD_600_ of approximately 0.35. Cephalexin (Dr. Ehrenstorfer GmbH, Augsburg, Germany) was added to the culture at a concentration of 1.0 μg/ml and cultured for 60 min. The control group was spiked with an equal volume of water without cephalexin. Six replicate samples were prepared, quenched by 45% ME and extracted with 75% BE, and then analyzed by UHPLC-TOF/MS.

### Untargeted metabolomics analysis.

The intracellular metabolites were analyzed using a UHPLC-TOF/MS (6600+; AB Sciex, Framingham, MA, USA). Metabolites were separated on both a reversed phase (RP) column and a hydrophilic interaction liquid chromatography (HILIC) column, with the oven temperature at 40°C, and detected in both positive and negative electrospray ionization (ESI) modes. For the RP separation, a Waters BEH Shield RP C_18_ column (2.1 by 100 mm, inside diameter [i.d.] of 1.7 μm) was used, and the mobile phases used were solvents A (0.1% formic acid in water) and B (0.1% formic acid in acetonitrile) for ESI^+^ mode and solvents A (5 mM ammonium acetate in water) and B (5 mM ammonium acetate in acetonitrile) for ESI^−^ mode. The linear gradient program was as follows: 0 to 1 min, 2% B; 2 to 10 min, 2 to 40% B; 10 to 11 min, 40 to 98% B; 11 to 12 min, 98% B; 12 to 12.1 min, 98 to 2% B; 12.1 to 15 min, 2% B. For the HILIC separation, a Waters BEH amide column (100 by 2.1 mm, i.d. of 1.7 μm) was used, and the mobile phases were solvents A (0.1% formic acid in water) and B (0.1% formic acid in acetonitrile) for ESI^+^ mode and solvents A (5 mM ammonium acetate in water) and B (5 mM ammonium acetate in acetonitrile) for ESI^−^ mode. The linear gradient program was as follows: 0 to 2 min, 95% B; 2 to 8 min, 95 to 70% B; 8 to 9 min, 70 to 50% B; 9 to 10 min, 50% B; 10 to 10.1 min, 50 to 95% B; 10.1 to 15 min, 95% B. The flow rate was 0.3 ml/min, and the injection volume was 2 μl. The triple-TOF/MS parameters were set as follows: curtain gas, 30 lb/in^2^; ion source, ± 4,500 V; ion spray probe temperature, 500°C; nebulizer gas (N_2_), 50 lb/in^2^; auxiliary heating gas (N_2_), 50 lb/in^2^; de-clustering potential, 80 eV. Stepped collision energies were set at 20, 35, and 50 eV. The mass range was set to 50 to 1,200 Da, operating in independent data acquisition mode.

### Data processing and analysis.

The raw data were acquired using Analyst Software (AB Sciex) and preprocessed for baseline correction, alignment, and peak picking using Progenesis QI v.2.3 (Nonlinear Dynamics, Newcastle, UK). The detected features were filtered (*P *< 0.05, coefficient of variation [CV] < 20%, variable importance in the projection [VIP] >1, fold change ≥ 1.5) to identify the differential metabolites caused by antibiotic perturbation. The metabolites were identified using database searches against HMDB (http://www.hmdb.ca/) and ECMDB (http://www.ecmdb.ca) or by metabolite standards (Alta Scientific Company, Tianjin, China). Multivariate analysis and visualization were performed using EZinfo software (Waters), MetaboAnalyst 5.0 (https://www.metaboanalyst.ca), and R (4.0.5 versions), including PCA and orthogonal partial least-squares discriminant analysis.

### Data availability.

The raw metabolomics data have been deposited in the MetaboLights repository under accession number MTBLS3222.
