# Supplementary material for: Improved Sample Preparation for Untargeted Metabolomics Profiling of Escherichia coli
Source: Microbiol Spectr. 2021 Oct 6;9(2):e00625-21. doi: 10.1128/Spectrum.00625-21 (PMC8510244; doi:10.1128/Spectrum.00625-21)
Supplement: Supplemental file 1 — Supplemental material. Download SPECTRUM00625-21_Supp_1_seq1.pdf, PDF file, 3.2 MB [file spectrum00625-21_supp_1_seq1.pdf]

Supporting Information

Improved sample preparation for untargeted metabolomics profiling of *Escherichia coli*

Dongyang Ye, Xiaowei Li, Chengfei Wang, Saiwa Liu, Liang Zhao, Jingjing Du, Jian Xu, Jing Li, Lu Tian, Xi Xia#

College of Veterinary Medicine, China Agricultural University, Beijing 100193, China

Table of Content

Figure S1……………………………………………………………………S2

Figure S2……………………………………………………………………S3

Figure S3……………………………………………………………………S5

Figure S4……………………………………………………………………S5

Figure S5……………………………………………………………………S6

Figure S6……………………………………………………………………S7

Figure S7……………………………………………………………………S8

Figure S8……………………………………………………………………S9

Figure S9……………………………………………………………………S15

Table S1……………………………………………………………………S16

Table S2……………………………………………………………………S19

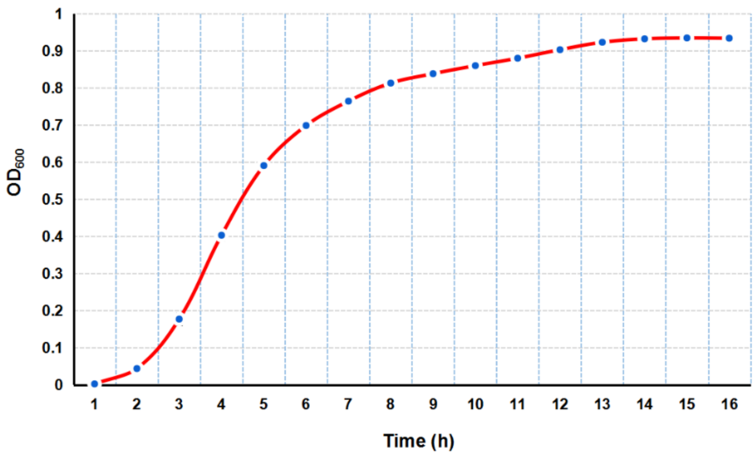

Figure S1. The growth curve of *E. coli* ATCC 25922. The OD_{600} was measured every hour. The mid-exponential phase of strain was determined according to the growth curve.

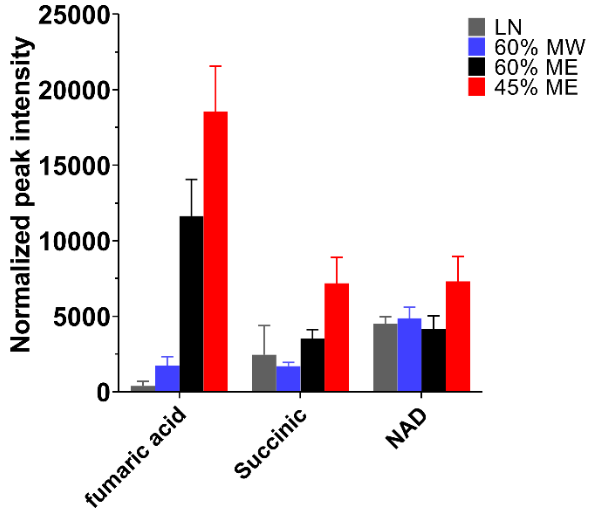

Oxidative phosphorylation

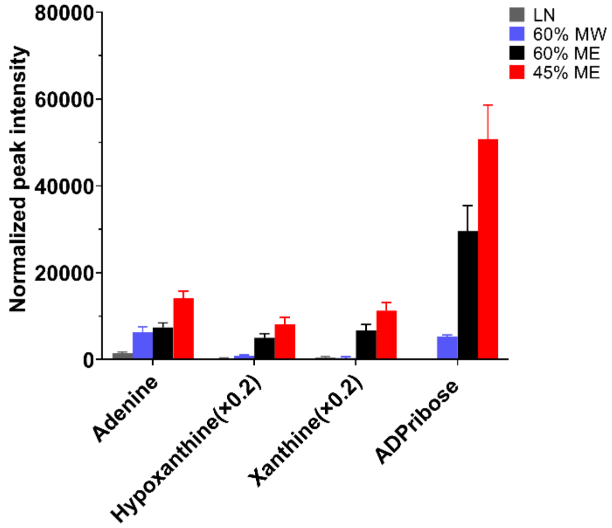

Purine metabolism

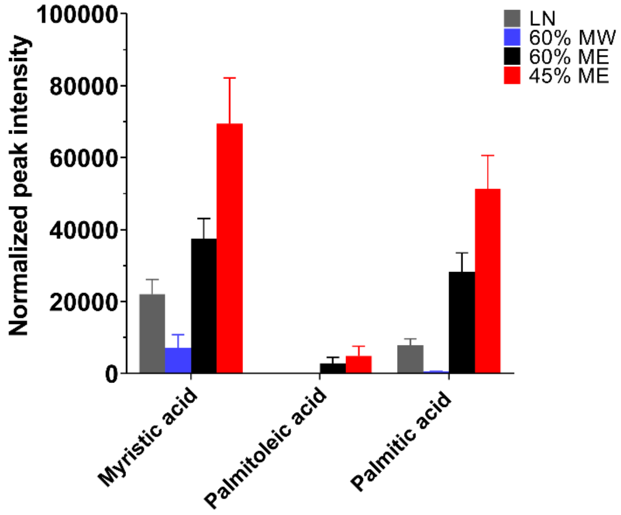

Fatty acid biosynthesis

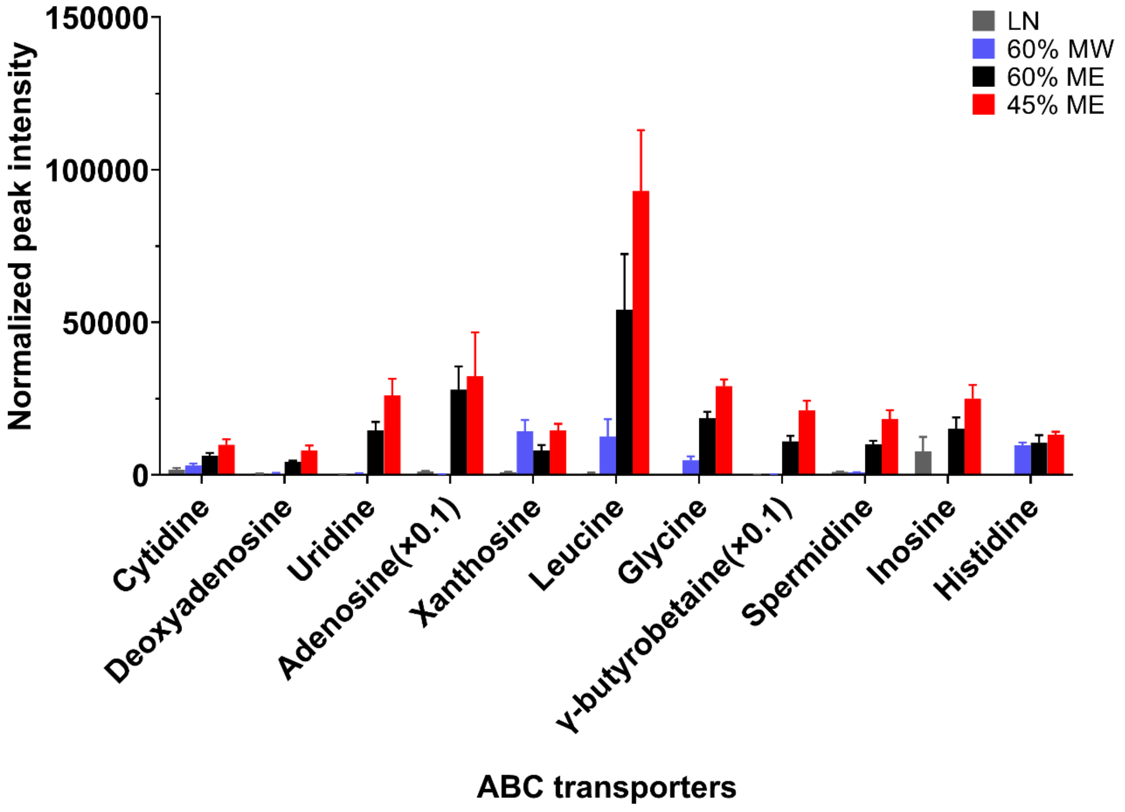

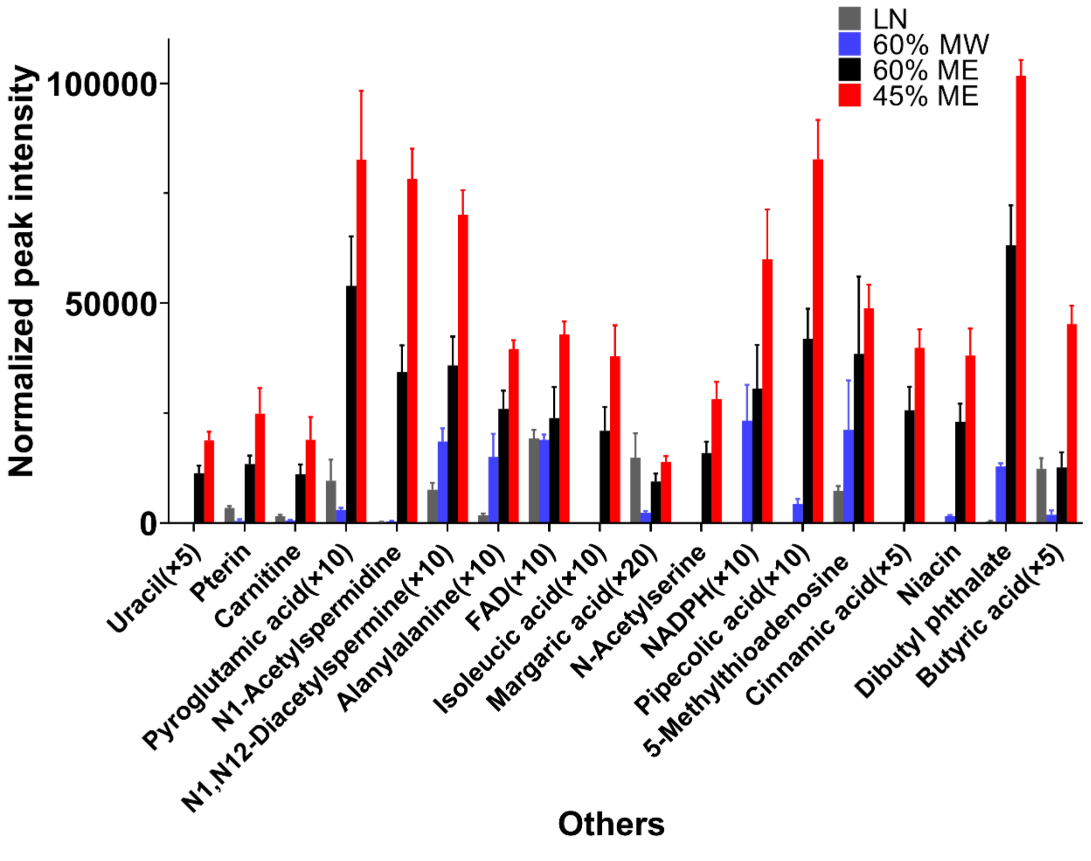

Figure S2. Abundance of representative metabolites from four quenching approaches.

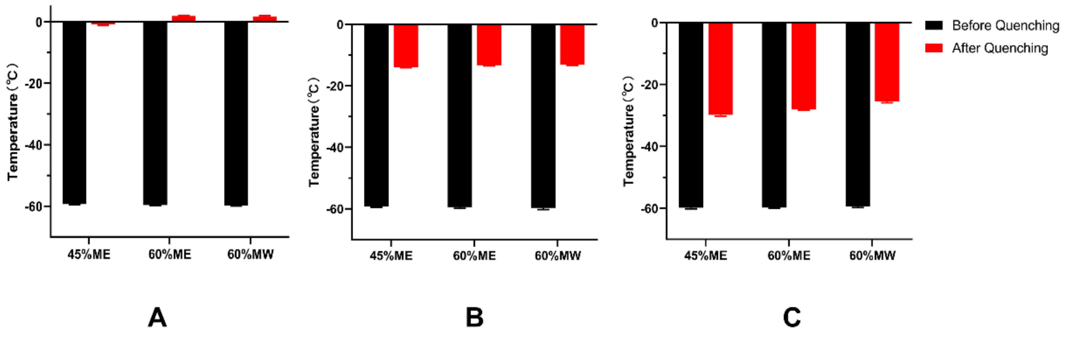

Figure S3. Effects of sample to quenching solvent ratio on quenching temperature. (A) Sample to quenching solvent at 1:2. (B) Sample to quenching solvent at 1:3. (C) Sample to quenching solvent at 1:4.

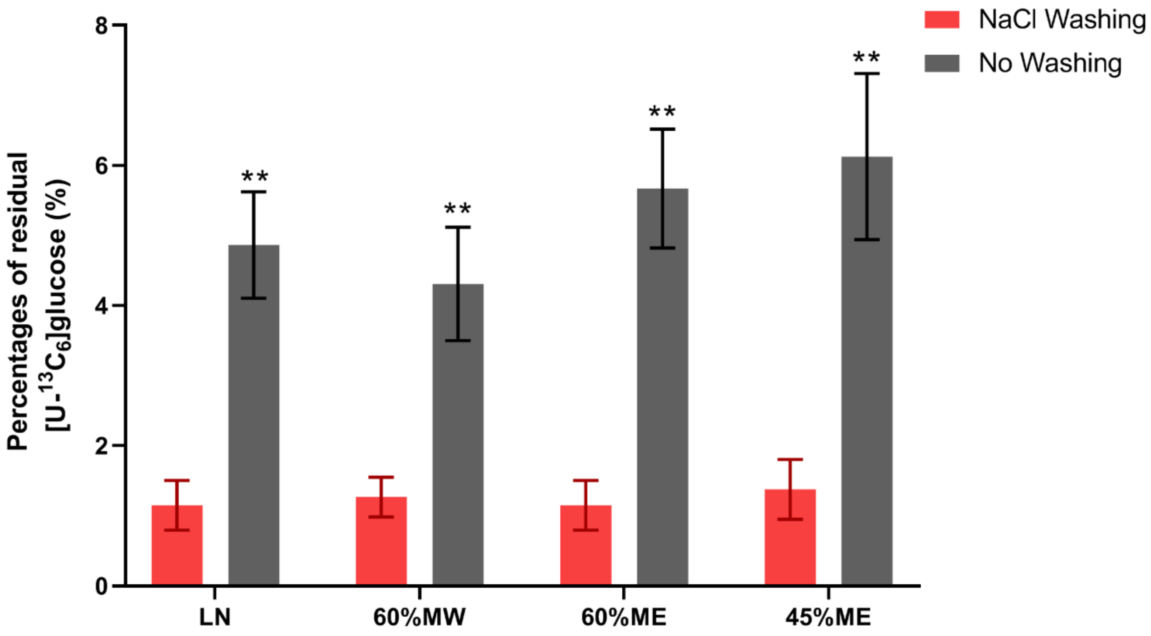

Figure S4. Percentage of residual [U-$^{13}C_6$]glucose after NaCl washing.

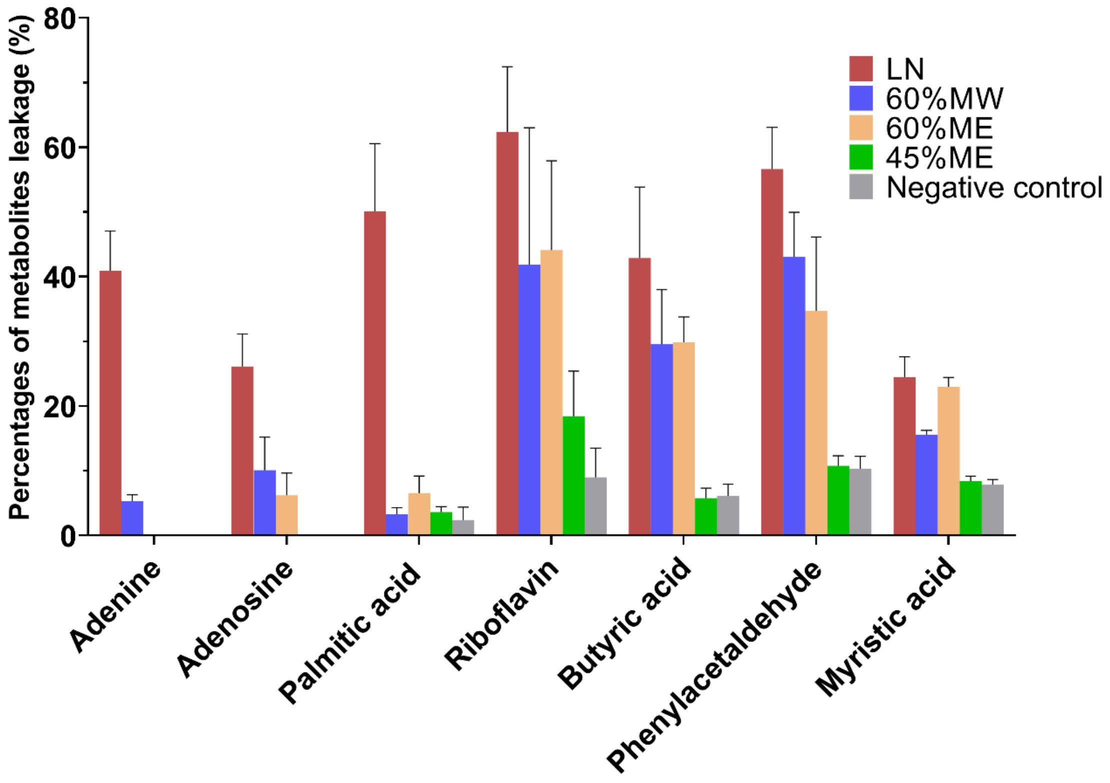

Figure S5. Leakage rate of intracellular metabolites after NaCl washing.

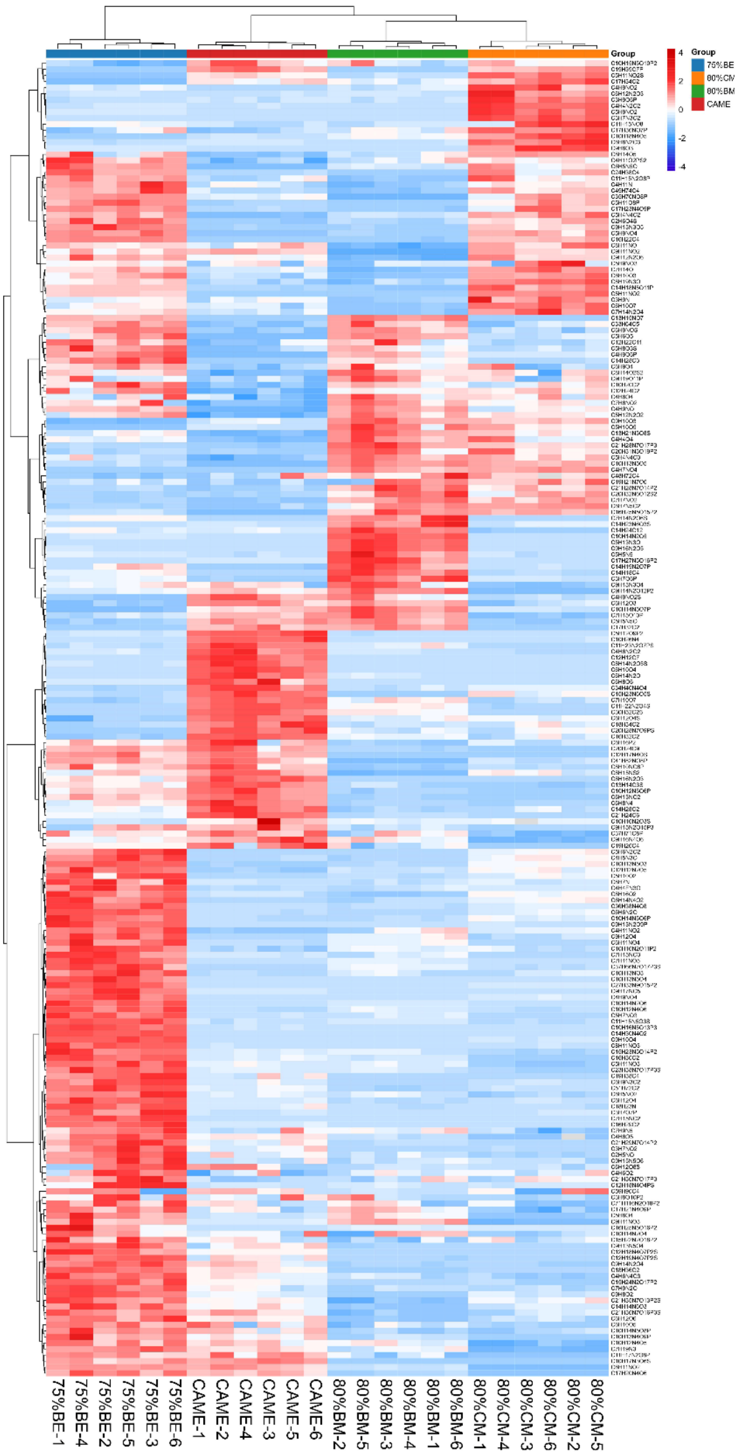

Figure S6. Hierarchical clustering analysis of intracellular metabolites from *E.coli* using different extraction solvents. Boiling ethanol/water (75% BE; 75:25, v/v, 95°C); cold methanol/water (80% CM; 80:20, v/v, -20°C); boiling methanol/water (80% BM; 80:20, v/v, 80°C); cold acetonitrile/methanol/ethanol/water (CAME; 20:20:20:40, v/v, -20°C).

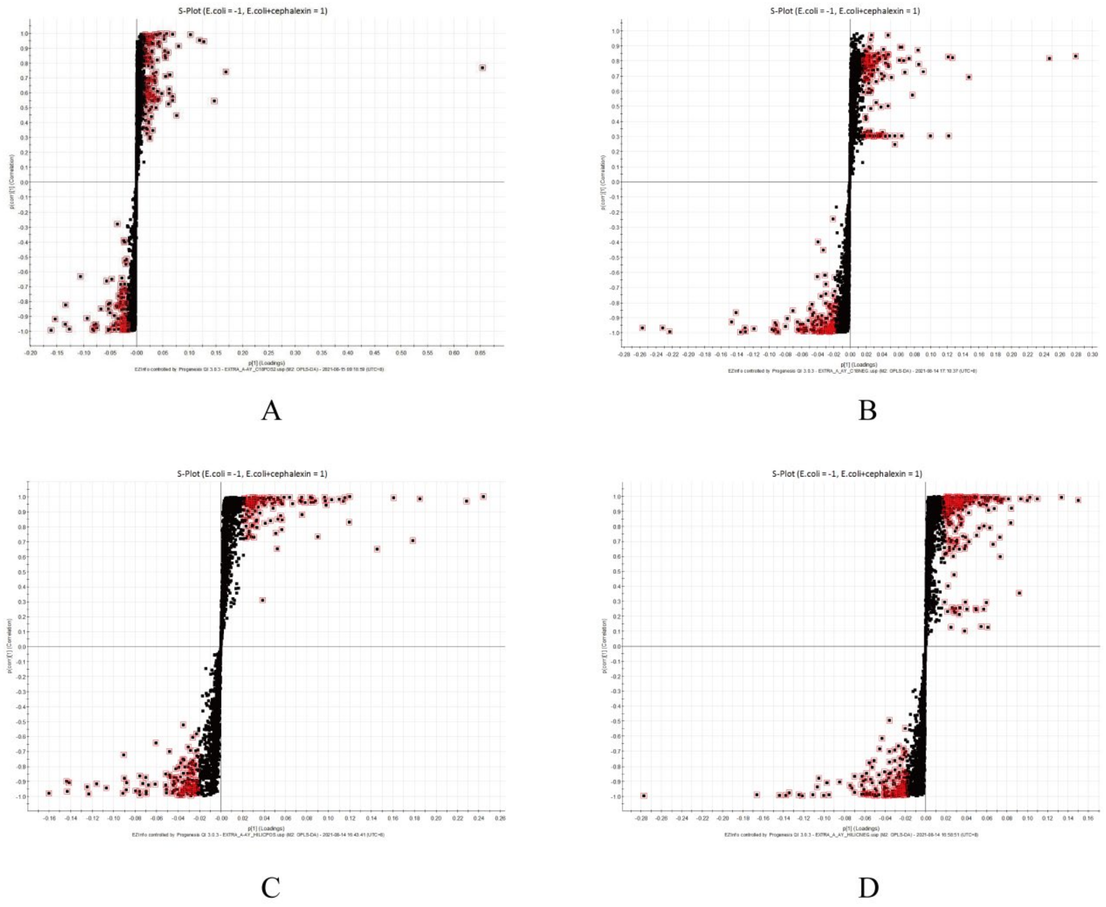

Figure S7. The S-plot of OPLS-DA in differential metabolites analysis. (A) C_{18}-positive mode; (B) C_{18}-negative mode; (C) Amide-positive mode; (D) Amide-negative mode. The red features: VIP＞1.

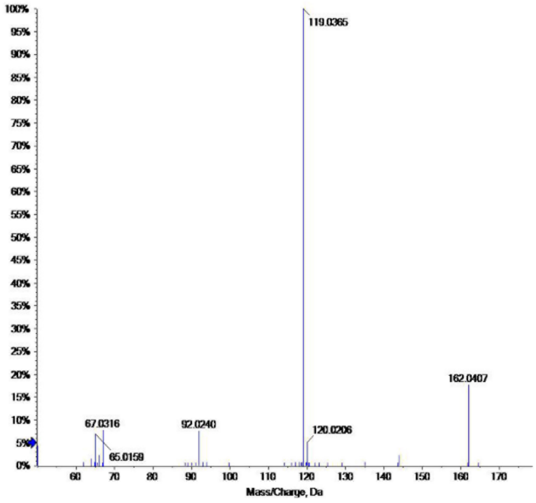

Sample

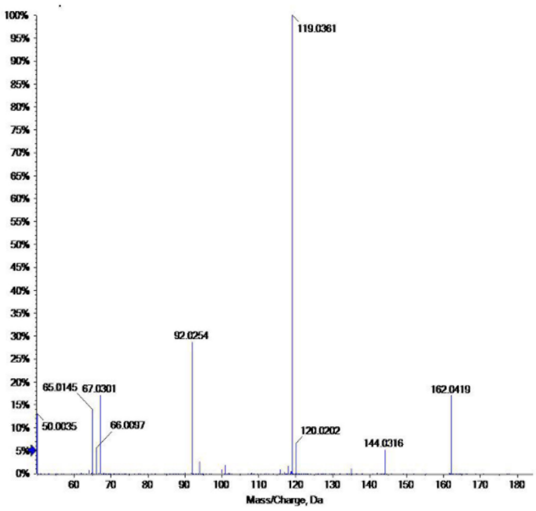

Standard

Pterin (M-H)

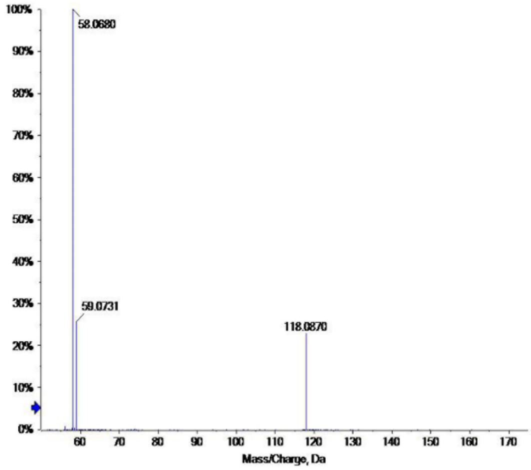

Sample

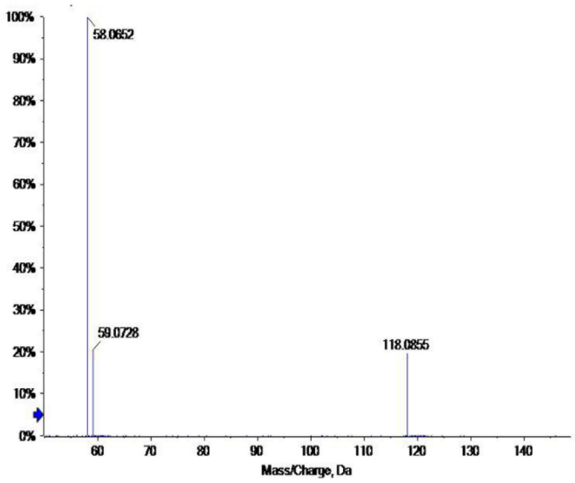

Standard

Glycine betaine (M+H)

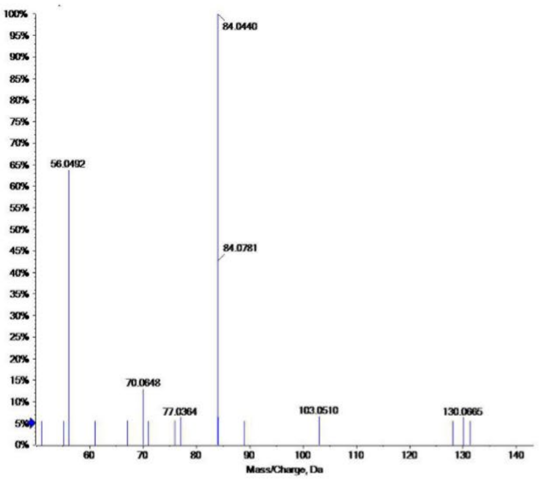

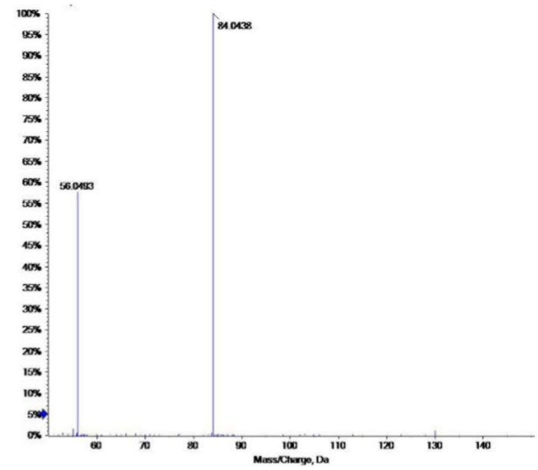

Sample **Standard**

Pyroglutamic acid (M+H)

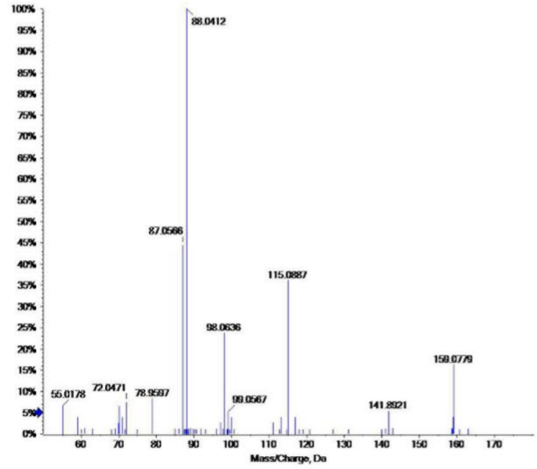

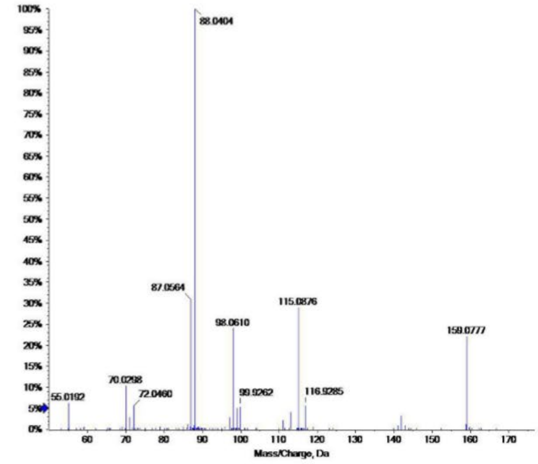

Sample **Standard**

Alanylalanine (M-H)

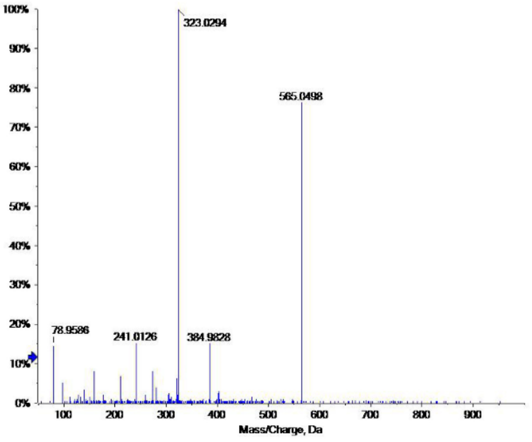

Sample

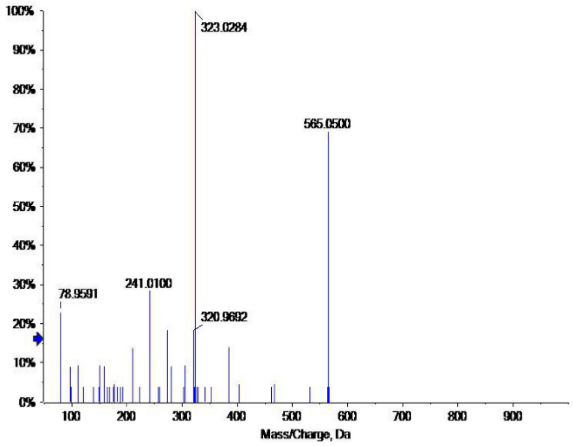

Standard

UDPglucose (M-H)

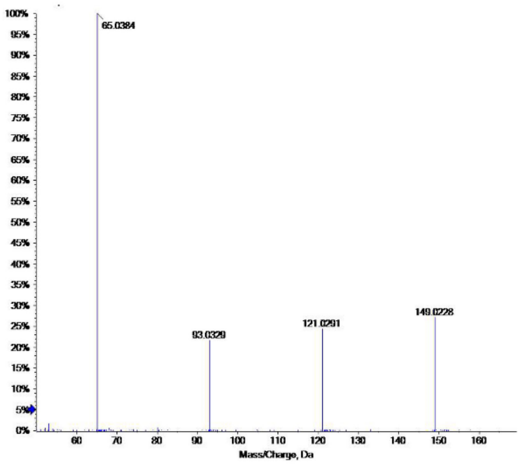

Sample

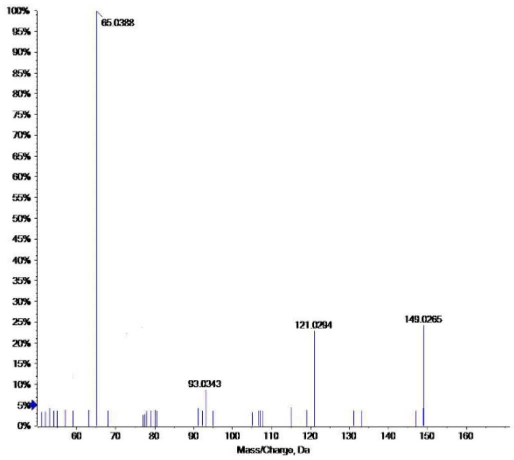

Standard

Cinnamic acid (M+H)

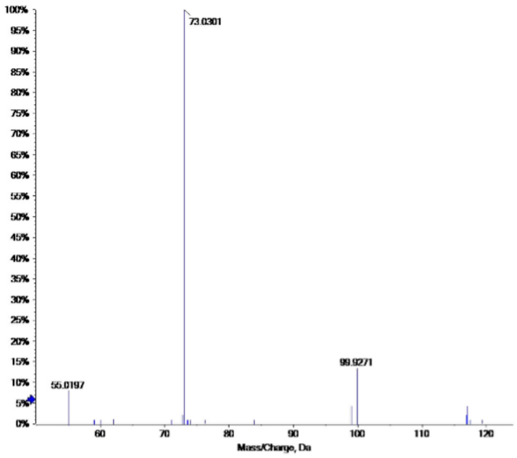

Sample

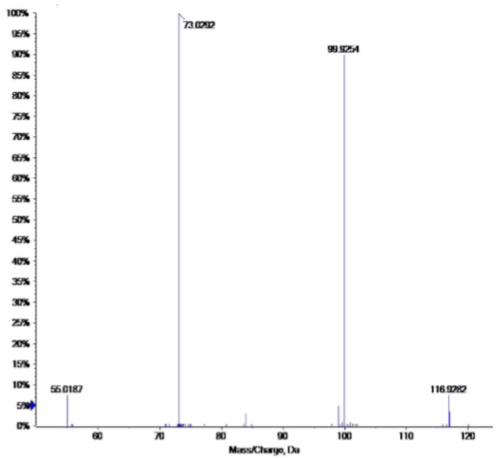

Standard

Succinic acid (M-H)

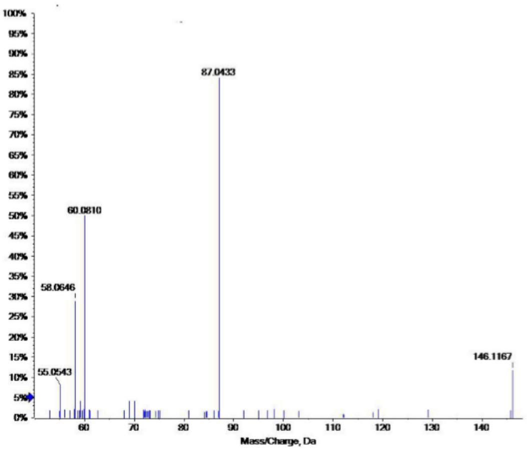

Sample

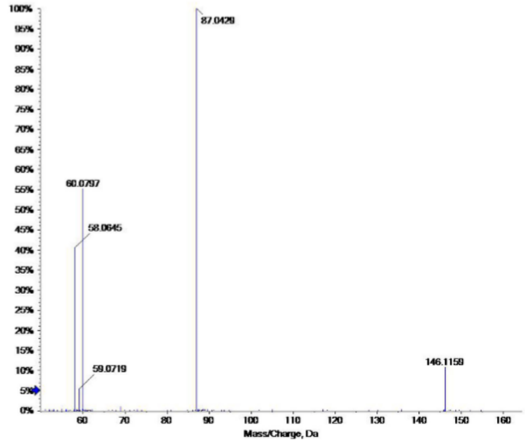

Standard

gamma-butyrobetaine (M+H)

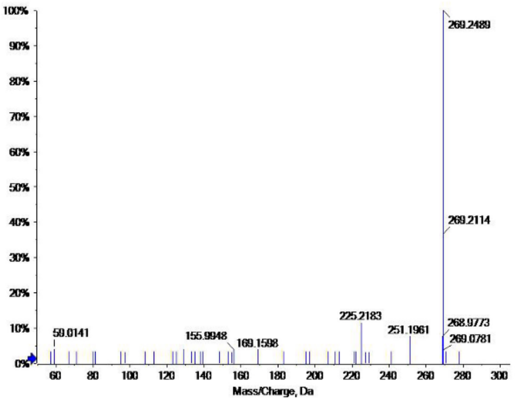

Sample

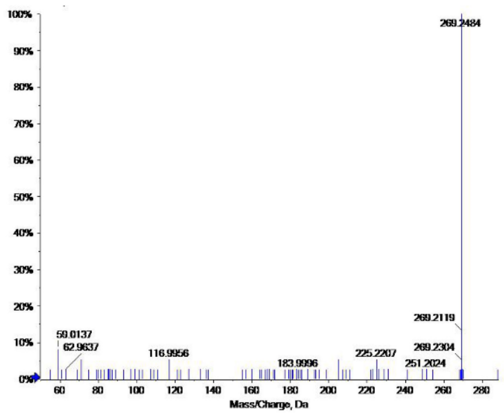

Standard

Margaric acid (M-H)

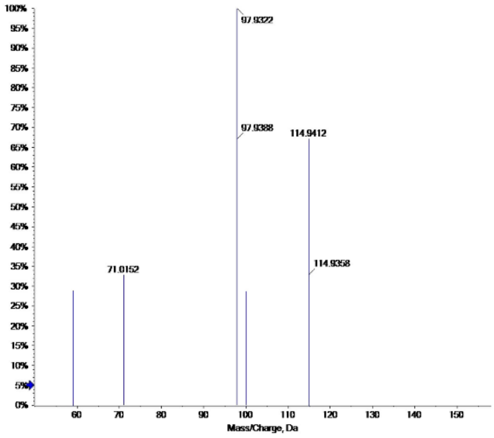

Sample

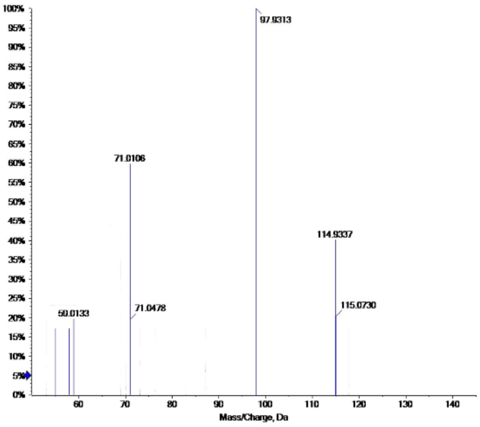

Standard

Fumaric acid (M-H)

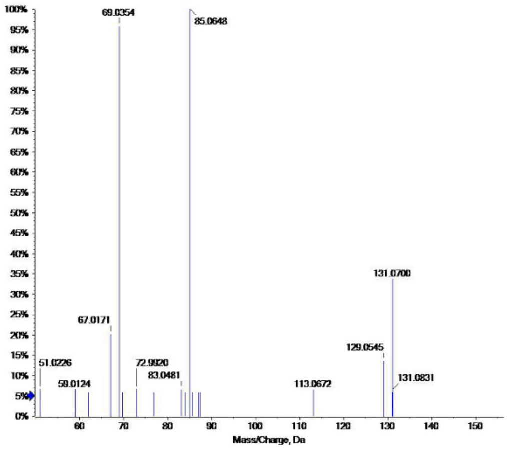

Sample

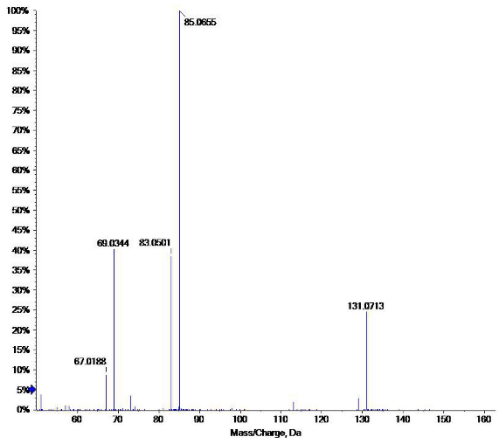

Standard

Isoleucic acid (M-H)

Figure S8. Intracellular metabolites identified using standards.

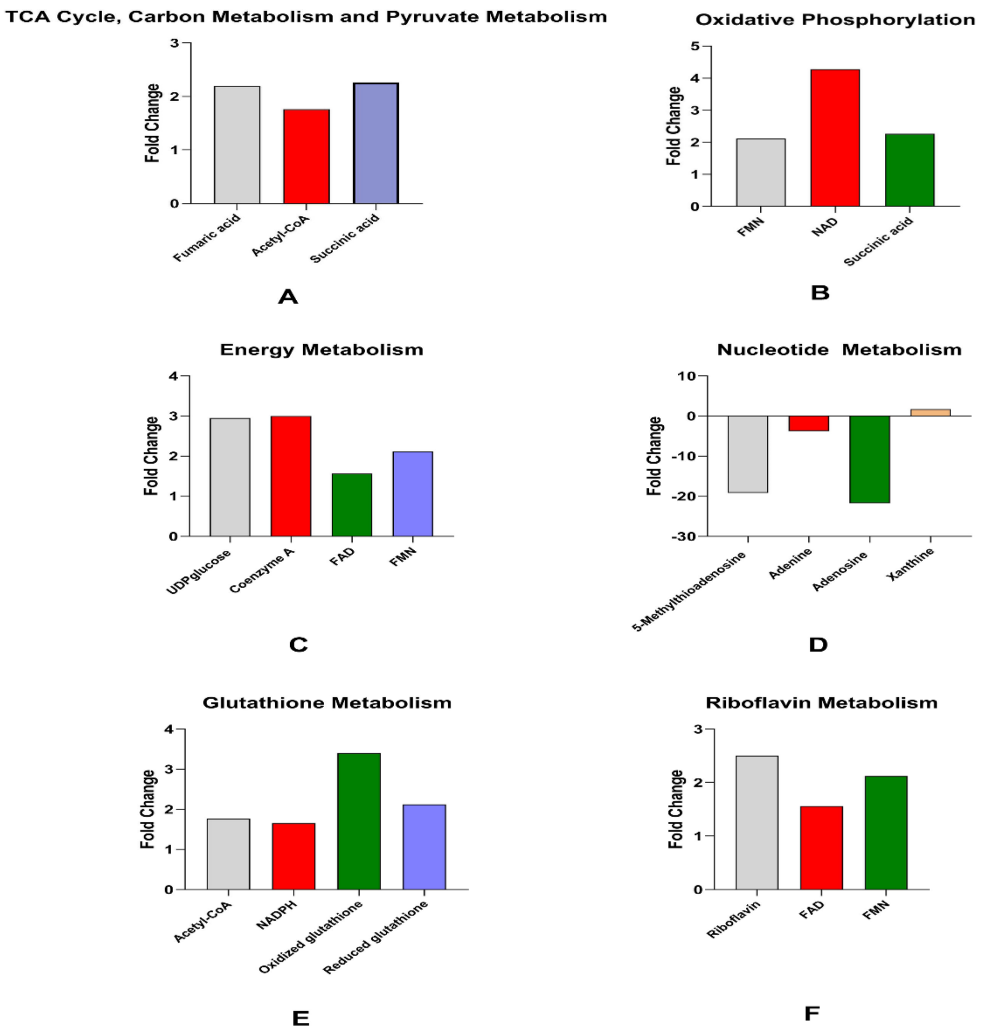

Figure S9. Specific pathway alterations induced by cephalexin.

Table S1 Metabolic perturbations of *E. coli* treated with cephalexin

Compound	Mode	Adducts	Formula	Compound ID	Anova (*P*-value)	Fold Change
**Upregulated**						
ADPribose	Amide NEG	M-H	$C_{15}H_{23}N_5O_{14}P_2$	C00301	2.11E-06	2.68
AMP	C_{18} NEG	M-H	$C_{10}H_{14}N_5O_7P$	C00020	2.15E-06	4.75
Acetyl-CoA	Amide NEG	M-H	$C_{23}H_{38}N_7O_{17}P_3S$	C00024	0.000337	1.75
Coenzyme A	Amide NEG	M-H	$C_{21}H_{36}N_7O_{16}P_3S$	C00010	3.34E-05	2.93
Cytidine	Amide POS	M+H	$C_9H_{13}N_3O_5$	C00475	3.36E-07	6.52
Alanylalanine	Amide NEG	M-H	$C_6H_{12}N_2O_3$	C00993	2.08E-07	4.03
Deoxyadenosine	Amide POS	M+H	$C_{10}H_{13}N_5O_3$	C00559	6.50E-11	2.13
FAD	Amide NEG	M-H	$C_{27}H_{33}N_9O_{15}P_2$	C00016	2.75E-05	1.53
FMN	Amide POS	M+H	$C_{17}H_{21}N_4O_9P$	C00061	0.000947	2.06
Glycine betaine	C_{18} POS	M+H	$C_5H_{12}NO_2$	C00719	4.47E-07	2.33
Hypoxanthine	Amide POS	M+H	$C_5H_4N_4O$	C00262	5.27E-05	1.73
Inosine	Amide NEG	M-H	$C_{10}H_{12}N_4O_5$	C00294	0.004320	1.65
Histidine	Amide NEG	M-H	$C_6H_9N_3O_2$	C00135	3.20E-07	3.16
Isoleucic acid	Amide NEG	M-H	$C_6H_{12}O_3$	HMDB0000317 ECMDB24038	4.47E-09	5.27
Leucine	Amide NEG	M-H	$C_6H_{13}NO_2$	C00123	0.000136	1.55
Margaric acid	C_{18} NEG	M-H	$C_{17}H_{34}O_2$	ECMDB21506	0.000265	2.95
Myristic acid	C_{18} NEG	M-H	$C_{14}H_{28}O_2$	C06424	1.87E-07	1.98
N1-Acetylspermidine	C_{18} POS	M+H	$C_9H_{21}N_3O$	C00612	0.001151	2.05
N-Acetylserine	C_{18} POS	M+H	$C_5H_9NO_4$	HMDB0002931 ECMDB21441	0.0019461	1.98

NADPH	C_{18} POS	M+H	$C_{21}H_{30}N_7O_{17}P_3$	C00005	0.000485	1.59
NAD	Amide POS	M+H	$C_{21}H_{27}N_7O_{14}P_2$	C00003	0.000194	4.28
Oxidized glutathione	C_{18} POS	M+H	$C_{20}H_{32}N_6O_{12}S_2$	C00127	3.22E-08	3.30
Pantetheine 4'-phosphate	Amide POS	M+H	$C_{11}H_{23}N_2O_7PS$	C01134	0.000592	2.45
Pipecolic acid	C_{18} POS	M+H	$C_6H_{11}NO_2$	C00408	0.000236	1.84
Reduced glutathione	Amide POS	M+H	$C_{10}H_{17}N_3O_6S$	C00051	0.002727	2.05
Riboflavin	C_{18} POS	M+H	$C_{17}H_{20}N_4O_6$	C00255	3.97E-05	2.45
Fumaric acid	Amide NEG	M-H	$C_4H_4O_4$	C00122	1.889E-05	2.14
Succinic acid	C_{18} NEG	M-H	$C_4H_6O_4$	C00042	1.34E-05	2.19
UDPglucose	Amide NEG	M-H	$C_{15}H_{24}N_{20}O_{17}P_2$	C00029	5.19E-08	2.88
5'-UMP	Amide POS	M+H	$C_9H_{13}N_2O_9P$	C00105	9.64E-05	2.17
Uridine	Amide NEG	M-H	$C_9H_{12}N_2O_6$	C00299	0.000590	1.91
Xanthine	C_{18} POS	M+H	$C_5H_4N_4O_2$	C00385	3.99E-05	1.70
Xanthosine	Amide NEG	M-H	$C_{10}H_{12}N_4O_6$	C01762	9.87E-06	1.56
**Downregulated**						
Pantothenate	Amide POS	M+H	$C_9H_{17}NO_5$	C00864	6.72E-05	-21.91
5-Methylthioadenosine	C_{18} POS	M+H	$C_{11}H_{15}N_5O_3S$	C00170	4.55E-07	-18.52
Adenine	Amide POS	M+H	$C_5H_5N_5$	C00147	4.46E-06	-3.75
Adenosine	Amide POS	M+H	$C_{10}H_{13}N_5O_4$	C00212	0.000116	-21.65
Cinnamic acid	C_{18} POS	M+H	$C_9H_8O_2$	C10438	3.28E-06	-4.79
gamma-butyrobetaine	C_{18} POS	M+H	$C_7H_{15}NO_2$	C01181	1.17E-05	-15.72
Carnitine	Amide POS	M+H	$C_7H_{15}NO_3$	C00318	0.000110	-19.86
Pyroglutamic acid	C_{18} POS	M+H	$C_5H_7NO_3$	C01879	5.73E-05	-4.07
N1, N12-Diacetylspermine	Amide POS	M+H	$C_{14}H_{30}N_4O_2$	C03413	3.83E-08	-12.84
Niacin	Amide POS	M+H	$C_6H_5NO_2$	C00253	9.88E-05	-4.31

Palmitoleic acid	Amide POS	M+H	$C_{16}H_{30}O_2$	C08362	0.006405	-8.62
Pterin	Amide NEG	M-H	$C_6H_5N_5O$	C00715	0.001083	-1.78
Pyridoxine	Amide POS	M+H	$C_8H_{11}NO_3$	C00314	2.70E-05	-1.76
Spermidine	Amide POS	M+H	$C_7H_{19}N_3$	C00315	5.07E-06	-2.30
Stearic acid	C_{18} NEG	M-H	$C_{18}H_{36}O_2$	C01530	1.34E-06	-2.86
Uracil	Amide POS	M+H	$C_4H_4N_2O_2$	C00106	9.01E-06	-2.55
Dibutyl phthalate	C_{18}POS	M+H	$C_{16}H_{22}O_4$	C14214	3.21E-09	-1.80

Table S2 Intracellular metabolites identified using MS/MS.

Compound	Mode	Matched fragment ions (*m/z*)							
ADPribose	NEG	558.0561	408.0111	346.0561	290.9686	211.0021	158.9258	96.9710	78.9588	
AMP	NEG	346.0530	150.9777	134.0454	107.0395	96.9706	78.9587			
Acety-CoA	NEG	808.1252	728.1573	461.0558	426.0224	408.0129				
FMN	POS	457.1125	439.1016	421.0918	359.1361	341.1235	243.0874	198.0678	172.0875	
Coenzyme A	NEG	766.1171	686.1566	437.0593	426.0231	408.0116	134.0482	78.9582		
Cytidine	POS	112.0496	95.0239	94.0380	69.0441					
Deoxyadenosine	POS	136.0611	119.0344	117.0558	99.0438	73.0283				
N1-Acetylspermidine	POS	188.1765	171.1495	112.1116	100.0759	72.0803	58.0648			
N-Acetylserine	POS	130.0506	102.0542	84.0439	56.0496					
Riboflavin	POS	377.1452	359.1332	243.0877	200.0813	198.0676	172.0873			
Xanthine	POS	153.0410	136.0139	110.0347	83.0245	82.0400	55.0290	53.0137		
5-Methylthioadenosine	POS	298.099	249.9998	145.0307	136.0618	119.0353	119.035	97.0287	75.0263	61.0102
Hypoxanthine	POS	137.0462	119.0356	110.0352	94.0402	82.0403	55.0296			
Reduced glutathione	POS	233.0593	179.0492	162.0228	142.0321	116.0158	84.0443	76.0215	58.9947	
Pantothenate	POS	202.1084	184.0987	160.0953	142.0865	103.0748	90.0551	72.0441	70.0291	67.0532
Adenosine	POS	136.0617	118.0870							
N1,N12-Diacetylspermine	POS	287.2387	172.8638	171.1471	112.1094	100.0749	98.9831	72.0446		
Niacin	POS	124.0419	80.0511	79.0427	53.0396	52.0214	51.0235			
Pyridoxine	POS	152.0747	134.0600	124.0760	94.0658					
Spermidine	POS	112.1125	84.0820	72.0808	58.0658					
Uracil	POS	113.0325	96.0105	95.0287	70.0307	69.9960	68.0159	52.0207		
NAD	POS	664.1163	542.0678	524.0579	428.0365	348.0701	232.0834	136.0617		
Carnitine	POS	162.1132	103.0391	102.0915	85.0285	60.0810	59.0733	58.0655	57.0339	

5’-UMP	POS	152.9095	113.0357	97.0284						
Adenine	POS	136.0618	119.0352	109.0514	94.0395	67.0206				
FAD	NEG	784.1530	517.0494	437.0868	408.0118	346.0546				
Inosine	NEG	267.0685	135.0307	108.0206						
Palmitoleic acid	POS	255.2320	97.1019	81.0700	71.0851	69.0710	57.0697	55.0543		
Oxidized glutathione	POS	613.1584	595.1453	484.1154	409.0839	355.0734	235.0200	231.0432	177.0329	130.0495
Pantetheine 4’-phosphate	POS	261.1264	243.1159	175.0549	184.0961	142.0862	132.0467	72.0447		
Pipecolic acid	POS	130.0873	84.0806	84.0451	70.0649	69.0558	68.0483	67.0564	58.0640	56.0497
Uridine	NEG	183.0423	153.0307	140.0355	111.0209	110.0254	82.0289			
Xanthosine	NEG	283.0684	151.0262	108.0213						
NADPH	POS	746.0109	729.0711	318.0362	302.0435	136.0606				
Dibutyl phthalate	POS	205.0848	149.0260	121.0291	93.0335	65.0385	57.0699			
ATP	POS	507.9990	428.0356	410.0263	330.0515	249.0526	136.0616			
ADP	POS	428.0430	348.0704	330.0561	232.0834	136.0613				
Palmitic acid	NEG	255.2324	237.2220	59.0141						
Leucine	NEG	130.0861	84.0840	82.0648	78.9555	66.0359				
Myristic acid	NEG	227.2067	209.1909	208.9805	85.0335	59.0147				
Stearic acid	NEG	283.2604	265.2482	239.1076	183.0036	59.0129				
Butyric acid	POS	71.0096	61.0282	53.0044						
Phenylacetaldehyde	POS	121.0625	93.0451	79.0526	77.0399	65.0345	55.0548	51.0221		
Histidine	NEG	154.0618	137.0356	93.0471	81.0444	67.0298				
